# Prevalence and Demographic Risk Factors of *Mycobacterium tuberculosis* Infections in Captive Asian Elephants (*Elephas maximus*) Based on Serological Assays

**DOI:** 10.3389/fvets.2021.713663

**Published:** 2021-11-02

**Authors:** Taweepoke Angkwanish, Hans J. C. M. Vernooij, Anucha Sirimalaisuwan, Pattara Charernpan, Mirjam Nielen, Victor P. M. G. Rutten

**Affiliations:** ^1^Department Biomolecular Health Sciences, Division Infectious Diseases and Immunology, Section Immunology, Faculty of Veterinary Medicine, Utrecht University, Utrecht, Netherlands; ^2^National Elephant Institute, Forest Industry Organization, Lampang, Thailand; ^3^Department Population Health Sciences, Division Farm Animal Health, Faculty of Veterinary Medicine, Utrecht University, Utrecht, Netherlands; ^4^Center of Excellence in Elephant and Wildlife Research, Faculty of Veterinary Medicine, Chiang Mai University, Chiang Mai, Thailand; ^5^National Elephant Research and Health Services, Department of Livestock Development, Bangkok, Thailand; ^6^Department of Veterinary Tropical Diseases, Faculty of Veterinary Science, University of Pretoria, Pretoria, South Africa

**Keywords:** Asian elephant, tuberculosis, prevalence, risk factors, ELISA, TB Stat-Pak, serology, latent class analysis

## Abstract

To address putative TB statuses of elephants and to identify and quantify potential demographic risk factors for TB, three ELISAs specific for different mycobacterial antigens (ESAT6, CFP10, MPB83) and the TB Stat-Pak assay were used as surrogate serological markers for TB infection in elephants. In view of the low number of animals of which the infected status could be confirmed (4 out of 708) Latent Class Analyses of TB serology test outcomes was used to predict the putative TB status of each of 708 elephants as positive (17.3%), inconclusive (48.7%), or negative (34%) when assessed on a population basis. Correlation between test performance of the individual assays was high between the ELISAs, but low with that of the TB Stat-Pak assay. Risk factors, assessed based on cut off values for each of the ELISAs determined by ROC analysis, included sex, BCS, age, working time, feed type, management system, camp size and region. Old age elephants were more likely to show a positive TB serology test outcome, than younger ones. Elephants working 7 h per day and the ones in good condition BCS (7–11) were less likely to be positive in TB serology testing. In addition, fewer animals in the large camp size (31–50 elephants) were found to be positive in ELISA tests, compared to elephants in the other camp sizes. In this study, the North region had the lowest percentages of elephants with positive TB test outcome, the West region and to a lesser extend the other regions showed clearly higher percentages of positive animals. Even though assays used in the present study have not been validated yet, results obtained showed promise as diagnostic or screening tests. For the diagnosis of animals suspected to be infected, the ELISA tests, once further optimized for the individual antigens, can be used in parallel. For screening of complete camps for presence or absence of infection, a single optimized ELISA test can be utilized.

## Introduction

Tuberculosis (TB) is a re-emerging infectious disease that can be transmitted within and between humans and animal species by transfer of mycobacteria of the *Mycobacterium tuberculosis* complex (MTBC). Its main representatives are *M. tuberculosis* (1), particularly infecting humans, and *M. bovis* hosted by a broad range of domestic and wild animals, as well as humans (2, 3). Infection of captive and wild Asian elephants (*Elephas maximus*) with *M. tuberculosis* has been shown worldwide (4–7). Transmission of *M. tuberculosis* from humans to elephants as well as other species and vice versa, is likely to happen in situations of close and prolonged contact and may occur *via* air, droplets, mucus and feces (1, 4, 6, 8–12). Transmission between domesticated and wild elephants may furthermore occur in direct contact at their interface (6, 13–15) and, because mycobacteria can survive for more than 45 days in the environment, shared facilities and resources may be major risk factors for infection with *M. tuberculosis*. Especially in countries in South East Asia, like Thailand where TB prevalence in humans is very high (16), zoo employees, including ground keepers and custodians show a high incidence of interferon gamma release assay or tuberculin skin test positivity, indicating (latent) infection, related to their socio-economic background, thus identifying them as potential risk factors for the animals in their care (14).

*Mycobacterium tuberculosis* infection in elephants is a chronic disease, and difficult to diagnose (17, 18). The gold standard for TB diagnosis, definitely confirming infection, is bacterial culture. However, since this assay has a very low sensitivity (i.e., a high false negative rate) (4, 19, 20) and can only be conducted in a very limited number of elephants, alternative diagnostic assay have to be considered. An Interferon Gamma Release assay (IGRA), the diagnostic method of choice in humans (21, 22), is available for elephants (23, 24), but not yet validated, as a diagnostic test, besides for large surveys it is cumbersome to use due to strict demands for sampling and limited testing facilities. In the present study, as an alternative, serological assays were assessed for discrimination between infected and non-infected animals (25), The ultimate goal of the current study was to contribute to the design of control and prevention strategies for *M. tuberculosis* infection and its transfer to and within Asian elephants.

Like for humans, stressful conditions such as poor sanitation, starvation, and high workloads are considered to be risk factors for tuberculosis in elephants (26, 27). Furthermore, other stressors like transportation, separation or isolation, and training could activate latent tuberculosis in elephants, aggravate active disease, and induce respiratory shedding of MTBC into the environment (4, 10, 12). Thus, potential risk factors considered to be associated with *M. tuberculosis* infection (14, 28, 29) include the infection status of humans in close contact with elephants, and a number of intrinsic and extrinsic factors like elephant age, body condition score (BCS), exposure to infected animals or shared enclosures, water/food resources, working activities and time, camp size, and feeding management.

The aim of the present study using ESAT-6, CFP-10, and MPB83, as putative correlates of infection, in enzyme-linked immunosorbent assays (ELISAs) and the Stat-Pak assay, was to: (1) estimate the prevalence of *M. tuberculosis* infection in the captive elephant population in Thailand predicted by Latent Class Analysis (LCA) (25); (2) assess cutoff values, sensitivity and specificity of elephant humoral immune responses to specific MTB complex antigens; and (3) using the predicted elephant's TB status to assess associations with demographic characteristics.

## Animals, Materials and Methods

### Elephants

The study was conducted on captive Asian elephants (*Elephas maximus*) in Thailand that were visited by veterinarians for registration and health care purposes as part of a mobile elephant clinic project (MEC) between 2004 and 2009. Of 1,500 serum samples collected in this cross sectional survey, a randomly selected subset of 708 samples (47%) from individual elephants at 124 tourist camps in six regions of the country (13 provinces) were used for serological diagnosis. This sample set represented ~18.7% of the captive elephant population in Thailand.

Elephant information provided by keepers and owners included: owner name/address; elephant name, age and sex; elephant ID; physical examination data; a description of the elephants habits/behavior; elephant current and past work places and daily routines; camp sizes; and feeding types and general management.

### Data Collection

Serum samples bank, obtained from routine health care at the National Elephant Institute (NEI), Lampang, Thailand was analyzed using the Stat-Pak assay or three different ELISAs. Elephants were categorized into five age groups: young (<3 years), sub adult (4–10 years), adult (11–50 years), middle age (51–60 years) and retired (over 60 years) as described by Corvanich (30). Working times were divided into four categories: no work (0 h), limited work (2 h/day), intermediate work (3 h/day) and full day work (7 h/day). Three feeding types were distinguished: natural feeding, provided (by man) feeding, and a combination of provided and natural feeding. Elephant management consisted of either intensive or extensive categories (31). The intensive management system is characterized by provided feeding, and prolonged times in proximity of and under the care of humans. With an extensive management system, elephants were more independent, and allowed to forage in the forest at night; hence, natural feeding is only supplemented by man. Elephant camp sizes were divided into four categories: small camps (1–9 elephants), medium camps (10–30 elephants), large camps (31–50 elephants) and very large camps (over 51 elephants). Body condition scores (BCS) based on visual observation of several body regions were categorized as poor (scores 1–2), thin (scores 3–4), optimal (scores 5–8) and obese (scores 8–11) (32).

### Serological Assays

The Elephant TB Stat-Pak, a lateral-flow test (Chembio Diagnostic Systems, Inc., Meldford, NY) employing specific antigens common to *M. bovis* and *M. tuberculosis* for the detection of antibodies was performed according to manufacturer's instructions (19, 33). For quantitative assessment of antibodies specific for individual MTBC related recombinant antigens, indirect ELISAs were conducted as follows. First wells of 96-well Microwell™ maxisorb ELISA plates (Nunc, C96 446140) were coated overnight at 4°C with 50 μl of a 1 μg/ml solution (in PBS, pH 7.4) of one of three recombinant antigens: early secreted antigen target 6 (ESAT-6); culture filtrate protein 10 (CFP-10); and cell surface lipoprotein MPB83. Subsequently, plates were washed once with 0.1% Tween20 in PBS (pH 7.4). Finally, 150 μl of a 0.1% blocking buffer solution (Cat. No. 11112589001, Roche™) in sterile water was added per well for 30 min at room temperature. After this blocking step, wells were washed once more and elephant sera added at a dilution of 1:800 in blocking buffer (50 μl/well). This dilution was shown to provide an optimal range in optical density (OD) signal between positive and negative control sera. Two control serum samples were analyzed in each plate: one from an elephant confirmed to be infected (positive control), and one from a healthy elephant in the Netherlands without a history of tuberculosis or contact with infected animals (negative control). After 60 min of incubation at room temperature, plates were washed three times with 0.1% Tween 20 in PBS (pH 7.4) after which 50 μl/well of rabbit anti-elephant IgG, diluted 1:8,000 in blocking buffer was added for 30 min at room temperature. Plates were washed three times again, followed by addition of 50 μl/well goat anti-rabbit IgG H+L (HRP) conjugate diluted 1:2,000 in 0.1% blocking buffer (KPL's HRP stabilizer cat. No. 54-15-01) and incubation for 30 min at room temperature, after which plates were washed five times again. Finally 50 μl/well of substrate [2,2′-azinobis 3- ethylbenzothiazoline-6-sulfonic acid (ABTS) tablets, Cat. No. 11112 422 001, dissolved in buffer (Cat. No. 11112597001), Roche®] prepared according to manufacturer's instructions was added and the OD (405 nm) read after 15 min.

ELISA values were expressed as sample to positive ratio [S/P ratio = (OD of sample – OD of negative control)/(OD of positive control – OD of negative control)] × 100.

### Statistical Analyses

The S/P ratios obtained in the ESAT6, CFP10, and MPB83 ELISAs as well as the positive/negative scores of the TB Stat-Pak assays were subjected to pairwise comparisons. The Pearson's correlation was calculated except for the TB Stat-Pak assays with ELISAs when Spearman rank correlation was applied. Latent Class Analysis (LCA) was applied using ESAT6, CFP10, MPB83, and TB Stat Pak assays to predict the three TB serology test outcomes (positive, inconclusive, negative) (34).

Determination of cut-off values, sensitivity and specificity for the three ELISAs were achieved in two steps: (1) an LCA model using 3 out of 4 tests, excluding the specific test itself, was used to predict the three TB serology test outcomes; and (2) Receiver Operation Curve analysis (35) was applied for each of the tests with the predicted *positive* vs. *not-positive* TB serology test outcomes as the true outcome. Subsequently, the defined cut-off values were used to dichotomize the S/P ratios of the tests and results were cross tabulated (**Table 3**) with the three predicted TB serology test outcomes. The numbers of positive tests for each serum sample were also cross tabulated (**Table 4**) with the predicted TB serology outcome. The same procedure was applied to assess cut-off values for *negative* vs. *not-negative* TB test outcomes. The predicted TB test outcomes were cross tabulated with demographic factors and univariable logistic regression was applied to assess the strength of association between predicted *positive* vs. *not-positive* (inconclusive and negative combined) statuses and the respective demographic factors. Multivariable logistic regression with all demographic factors with backward elimination based on Akaike's Information Criterion (AIC) was applied to assess adjusted odds ratios. The analyses were done using the statistical program R (36) version 3.6.0.

## Results

Test results of CFP10 and MPB83 ELISAs of 708 elephant samples in the cross-sectional study showed high correlation (>0.6). In contrast pair wise correlations of these ELISAs with results of the ESAT-6 ELISA were moderate, whereas correlation of results of each of the three ELISAs with those of the commercial TB Stat-Pak assay were very low (Supplementary Table 1). Based on LCA, the proportions of the population of elephants in the predicted serological TB positive, inconclusive, and negative TB serology test groups were 17.3, 48.7, and 34%, respectively (Tables 1, 2). In the predicted positive group, only 18.8% of the animals was TB Stat-Pak positive. The mean and standard deviation for S/P ratios resulting from the ESAT6, CFP10, and MPB83 ELISAs decreased from predicted positive to predicted negative TB serology test outcome (Table 2). Estimated cut-off values between *positive* and *not-positive* (negative and inconclusive combined) of each of the serological tests, determined using LCA, and corresponding sensitivity and specificity based on Receiver Operation Curve (ROC) analyses (see Supplementary Figure 1) are presented in Table 3. The sensitivity (Se) and specificity (Sp) measures were lowest for the ESAT6 ELISA, whereas Se was highest for the MPB83 ELISA and Sp was highest for the CFP10 ELISA. In addition, a second cut-off value was calculated to discriminate between TB serology test outcomes predicted as *negative* and *not-negative* (positive and inconclusive combined) predicted TB statuses. For CFP10 particularly, both cut-off values were relatively similar, the histogram of CFP10 shows a very narrow peak hence a little shift to the right has a huge effect (Supplementary Figure 2). For the other assays the cut-off value was clearly lower for the second approach. Comparing the TB serology test outcomes predicted as *negative* vs. *not-negative* (inconclusive and positive) similar values for sensitivity (for *not-negative* test) and specificity (for *negative* test) are observed except a much lower Se for CFP10.

**Table 1:** Percentages of elephants (*n* = 708) per LCA predicted per TB serology test outcome and concordance with the TB Stat-Pak assay outcomes for each of the TB statuses.

	**Predicted TB serology test outcome**					
	Positive (17.3%)^*^		**Inconclusive (48.7%)**		**Negative (34%)**	
**TB Stat-Pak**	**Pos**.	**Neg**.	**Pos**.	**Neg**.	**Pos**.	**Neg**.
Proportion	18.8%	81.2%	3.9%	96.1%	1.4%	98.6%

* *Estimated population prevalence of TB serology test outcomes by applied LCA model*.

**Table 2:** Percentages of elephants (*n* = 708) per LCA predicted TB serology test outcome and concordance with the S/P ratios (mean and standard deviation) resulting from the three ELISAs for each of the TB statuses.

	**Predicted TB serology test outcome**					
	Positive (17.3%)^*^		**Inconclusive (48.7%)**		**Negative (34%)**	
**S/P ratio**	**Mean**	**SD**	**Mean**	**SD**	**Mean**	**SD**
ESAT6	31.92	23.04	19.37	14.27	6.98	6.54
CFP10	41.63	17.77	20.04	8.29	7.50	5.41
MPB83	50.41	21.54	26.30	12.03	9.94	7.99

* *Estimated population prevalence of TB serology test outcomes by applied LCA model*.

**Table 3:** Receiver operation curve analysis: estimated cut-off value and subsequent sensitivity and specificity for each ELISA test based on the LCA predicted TB serology test outcomes.

**ELISA Test**	**Cut-off** 1^*^	**Sensitivity 1** **(Se1)**	**Specificity 1** **(Sp1)**	**Cut-off** 2^**^	**Sensitivity 2** **(Se2)**	**Specificity 2** **(Sp2)**
ESAT6	18.83	61.5%	68.2%	11.21	63.4%	62.8%
CFP10	21.15	81.4%	75.7%	18.87	55.4%	77.7%
MPB83	28.39	88.0%	71.3%	21.22	83.4%	75.3%

* *Cut-off value (S/P ratio) for predicted positive vs. not-positive TB serology tests at maximum sum of sensitivity and specificity per ELISA test, based on Latent Class Analysis with the two other ELISA tests and the TB Stat-Pak test*.

** *Cut-off value (S/P ratio) for predicted not-negative vs. negative TB serology tests at maximum sum of sensitivity and specificity per ELISA test, based on Latent Class Analysis with the two other ELISA tests and the TB Stat-Pak test*.

Test results were dichotomized based on the comparison *positive* vs. *not-positive* tests in the ROC analysis (positive: S/P ratio > cut-off). In the group of elephants with the predicted positive TB serology tests, the percentage of positive ELISAs was higher than 71%, whereas the TB Stat-Pak was positive for 23% of the animals in this group (Supplementary Table 2). The percentages of positive ELISAs in the group of elephants with inconclusive TB serology test outcomes were between 13.5% (CFP10) and 48.3% (ESAT6), whereas percentages were close to zero in the group with negative TB serology test outcomes, except for the ESAT6 ELISA which scored 21% positive. The TB Stat-Pak assay was positive in 4 and 2% of predicted inconclusive and negative test samples, respectively. The proportion of negative ELISAs in elephants with predicted positive TB serology test outcomes was between 13 and 28%, in case of animals with an inconclusive TB serology test outocmes it was higher than 52%, and higher than 79% in those with negative TB serology tests. The TB Stat-Pak assay scored negative in more than 77, 96, and 98% in animals with predicted positive, inconclusive and negative TB serology testoutcomes, respectively.

Most samples (94%) with a predicted negative TB serology test outcome were negative in all four single serological tests (Table 4). In the group with predictive inconclusive TB serology test outcomes, about 59% of the samples had none or only one single test positive. In contrast, in the group of elephants with a positive predicted TB serology test outcome, 97% of the samples were positive in two to four serological tests.

**Table 4:** Number of positive serological tests (out of 4) done (ESAT6, CFP10, MPB83 and TB Stat-Pak) of elephant samples vs. the predicted TB serology test outcome based on latent class analysis.

		**Predicted TB serology test outcomes**		
	**Total**	**Positive** **105 (15.0%)**	**Inconclusive 357 (50.4%)**	**Negative 245 (34.6%)**
**Number of positive serological tests**	**n (%)**	**n (%)**	**n (%)**	**n (%)**
0	271 (38.3)	0 (0)	42 (11.8)	229 (93.5)
1	186 (26.3)	3 (2.8)	167 (46.8)	16 (6.5)
2	142 (20.1)	36 (34.0)	106 (19.9)	0 (0)
3	90 (12.7)	51 (48.1)	39 (10.9)	0 (0)
4	19 (2.7)	16 (15.1)	3 (0.8)	0 (0)

*n, number of animals; %, percentage within predicted TB serology test outcome*.

The distributions of the three predicted Tb serology test outcomes within the 708 individual elephants across the categories of each demographic variable is presented in Supplementary Table 3. Though actual numbers are low elephants with a high BCS (7–8 and 9–11) were more often serologically negative than elephants with lower BCSs. The proportion of elephants predicted to be TB serology test positive tended to increase with age. The proportion of elephants with negative predicted TB serology test outcomes was highest in the North region. For the other demographic variables representation of the three predicted TB serology test outcomes did not differ between categories.

Table 5 shows the results of the logistic regression modeling for each of the 8 demographic variables. Odds of elephants of both sexes having positive predicted TB serology test were similar. Individuals with a BCS higher than 6 were significantly less likely to have a positive predicted TB serology test compared to the 5–6 group. Older elephants (>50 years) were significantly more likely to have a positive predicted TB serology test than 10–50 years old elephants. Animals with a workload of 7 h were significantly less likely to have a positive predicted TB serology test compared to those with a workload of 0–3 h per day. Elephants in the North region were significantly less likely to have a positive predicted TB serology test than in the other regions (West, South and Central, East, and Northeast, respective). Elephants from large camps (31–50) were significantly less likely to have a positive predicted TB serology test outcome compared to those in small camps (1–9). Differences in sex, feed type and management system did not show association with predicted serological TB serology test outcomes.

**Table 5:** Estimates of the strength of association (odds ratio and 95% confidence interval) for positive TB serology test outcome with demographic factors based on univariable logistic regression models per factor.

			**Confidence Interval**	
**Variable**	**Category**	**OR**	**2.5%**	**97.5%**
Sex	Female (Ref)	1		
	Male	1.1	0.7	1.7
BCS	1-2, 3-4	1.1	0.4	2.5
	5-6 (Ref)	1		
	7-8, 9-11^*^	0.2	0.01	0.9
Age category (y)	1-9	0.8	0.4	1.6
	10-50 (Ref)	1		
	51-100^*^	2.3	1.1	4.7
Working time	0-3 (Ref)	1		
(hours/day)	7^*^	0.4	0.2	0.8
Feed type	Natural-Provided (Ref)	1		
	Natural	1.3	0.7	2.3
	Provided	1.3	0.8	2.2
Management system	Extensive	1		
	Intensive	1.2	0.8	1.9
Camp size (n)	1-9 (Ref)	1		
	10-30	1.0	0.6	1.7
	31-50^*^	0.3	0.1	0.6
	>51	0.7	0.4	1.3
Region	North (Ref)	1		
	Central, East, Northeast^*^	2.3	1.2	4.7
	South^*^	3.8	2.0	7.4
	West^*^	7.4	4.0	14.3

*OR, odd ratio*.

* *Significantly different from reference category*.

**Figure 1:**
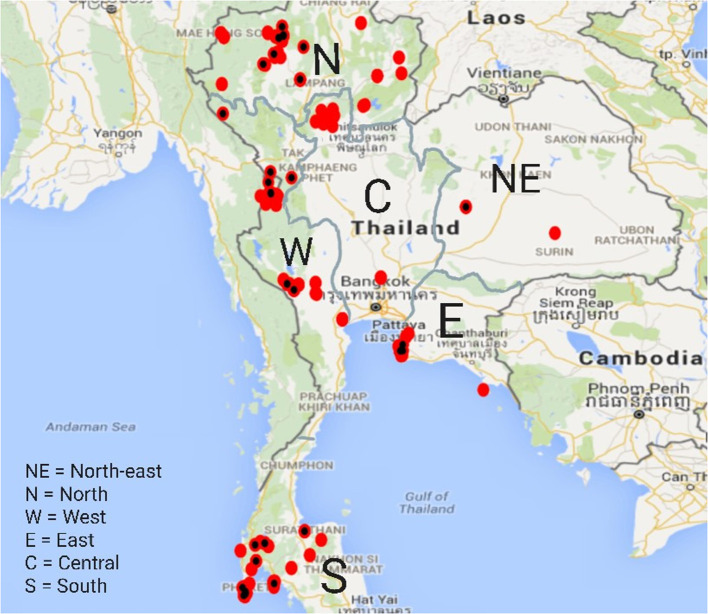
Spatial distribution of elephants in the study across Thailand. Red dots marked with a black spot indicate presence of at least one elephant to be of the predicted positive TB serology test outcomes.

After reduction of the multivariable logistic regression model with all variables, only “region” remained in the final model, indicating likely confounding between “region” and the management variables. The spatial distribution of the elephants at time of sampling is presented in Figure 1. Elephants are not evenly distributed across Thailand but regionally concentrated in mainly touristic areas.

## Discussion

In the present study four different serological assays were assessed for their capability to discriminate between putative *M. tuberculosis* infected, seropositive for MTBC antigens, and non-infected (seronegative) animals (25). By Latent Class Analysis (LCA), their ability to predict the serological TB test outcome of each elephant was assessed, which resulted in groups of elephants qualified as predicted positive, inconclusive and negative, in TB serology test outcomes, respectively. Since none of the serology tests used, is so far is validated for diagnosis TB in elephants, they can only be considered as surrogate classifiers. Conducting the analysis of all tests assuming that the prevalence of animals with “predicted positive TB serology test outcome” reflects that of *M. tuberculosis* infected elephants the prevalence is at least 15% and including animals classified as inconclusive up to 65% of elephants may be infected.

Correlations between test performance of the individual assays was modest to high between the three ELISAs, the latter especially between those employing MPB83 and CFP10 as antigens. It was low between the TB Stat-Pak assay and the ELISAs. Samples of animals predicted to be of a positive serological TB status (17.3 % of the total population tested) showed highest S/P ratios in the MPB83 and CFP10, and less so in the ESAT6 ELISAs. In the predicted TB serology test inconclusive group (49%), respectively, the predicted negative group (34%) S/P ratios clearly tended to decrease. Hence, to predict negative TB statuses, CFP10 and MPB83 ELISAs are most useful. To predict a positive TB status most accurately parallel interpretation of the ELISAs detecting MPB83, ESAT6, and CFP10 specific antibodies (20, 37) is required. By contrast, the TB Stat-Pak test scored positive for only 23% of animals of the predicted positive serological TB status. Although the decreasing trend (positive vs. inconclusive vs. negative) was similar to that observed in the ELISAs, the TB Stat-Pak assay seemed more discriminatory between positive and the other predicted TB statuses (20).

Taken together, results from the three ELISAs and the TB Stat-Pak assay suggest that these tests can be utilized to manage TB infection risks in areas with limited laboratory capacity. Clearly further optimization of the ELISA's for use in the field especially in view of “true” specificity and sensitivity is needed but not easy to realize not knowing true TB statuses (38). Likewise inclusion of additional selected antigens may be useful, but not easy. In view more advanced test systems, with a multitude of antigens, like in the ENFER multiplex assay (39) may be highly sensitive and specific, but too complicated to conduct in the field. Obviously the IGRA still the test of choice in humans (21, 22) is available for elephants (23, 24) but not yet validated as diagnostic tool, besides for large surveys it is cumbersome to use due to strict demands for sampling and limited testing facilities. Still conducting Elephant IGRA as well as IGRA or Mantoux tests in humans in close contact with the elephants could shed more light on the overall TB status of e.g., an elephant camp. For diagnostic confirmation of animals suspected to be infected, we suggest to utilize the 4 tests in parallel, where at least 2 positive results would support high probability of infection (see Table 4). For the situation of monitoring the presence/absence of infection in camps, we suggest to test all animals with a single test, preferentially the CFP10-ELISA. When all animals test negative, the likelihood of infection in that group is very low. Management might focus on prevention of disease introduction into the group. If multiple animals test positive, further identification of the infected animals is warranted, and management of individual animals might be adapted to prevent transmission and subsequent clinical disease.

To manage infectious diseases, it is important to know which factors are associated with higher or lower risks of infection. Single demographic variables BCS, age, working time, camp size and region were associated to predicted serological TB statuses, although in a multivariable analysis only region remained associated. Elephants with a high BCS (7–11) appear to be at low risk for TB, as compared to low BCS elephants. Low BCS may be associated with poor management, which can in turn predispose elephants to sensitivity to *M. tuberculosis* infection (26, 40). Likewise old aged elephants were more likely to be positive in serological tests, potentially due to a long lifespan hence prolonged contact with infected elephants (41), and/or caretakers potentially infected with *M. tuberculosis*. (1, 11, 14, 16, 42–44). The finding that the western region of Thailand had a high percentage of elephants positive in serological tests, coincides with the highest incidence of human TB in Thailand (45, 46). Finally, cellular immunity that controls *M. tuberculosis* infection may wane during progression of infection and with age and be (47), switch to more prominent humoral immunity is a potential reason why older elephants were more likely to be positive in TB serology. Elephants working a full 7 h each day were at lower risk for TB as compared to elephants that partook in no or limited work activities. Exercise is important for mental and physical health (27, 48), where elephants participating in work activities showed better body condition and metabolic health than those that did not work (49). Alternatively reverse causation could also explain this association, with healthy animals with good BCS being able to carry out full working days.

Elephants in large camps (over 30 elephants) were more likely to be negative in serology. In general, larger camps have been in operation longer and are more likely to have standardized management protocols, including sufficient land, nutritious food, clean water, appropriate works loads, good sanitation, and often an on-site veterinarian (49, 50). In addition, elephant numbers in large camps are more stable whereas the animals living there have limited contact to other elephants or keepers from outside, as compared to those in camps of smaller sizes, mostly also due to better economic support. Assessment in comparison to camps of smaller sizes, mostly also due to better economic support. Assessment of other factors in the study, including sex, feed type, and management system did not reveal any differences in TB risk. Some factors like feed type and management systems, are closely related, resulting in uncertainty in interpretation. Risk factor results can be generalized to the population of elephants in tourist industry of which the elephants tested were representatives.

## Conclusion

The seroprevalence for MTBC of elephants in Thailand is at least 15%, possibly up to 65% if inconclusive results are included. Hence, in order to reduce the risk of transmission amongst elephants and from elephants to humans and other species, and vice versa, control is needed. Unfortunately, no manageable risk factors could be identified in the current study, as a seropositive TB status was related to region and age. Even though assays used in the present study have not been validated yet, results obtained showed promise for their use in the field. Especially when further optimized for the individual antigens and used in parallel interpretation, identification of *M. tuberculosis* infected elephants seems valid, while screening of camps can be carried out with a single optimized test.

## Data Availability Statement

The original contributions presented in the study are included in the article/Supplementary Material, further inquiries can be directed to the corresponding authors.

## Ethics Statement

Ethical review and approval was not required for the animal study because this work was a retrospective study, performed by analyzing the diagnostic samples from the serum bank, obtained during routine health controls by Mobile Elephant Clinic and the Elephant Hospital of the National Elephant Institute (NEI), Thailand. Therefore, the ethical approval was not applicable from the Animal Use Committee. Written informed consent was obtained from the owners for the participation of their animals in this study. Written informed consent was not obtained from the individual(s) for the publication of any potentially identifiable images or data included in this article.

## Author Contributions

All authors listed have made a substantial, direct and intellectual contribution to the work, and approved it for publication.

## Funding

This work was partially funded by Faculty of Veterinary Medicine, Chiangmai University.

## Conflict of Interest

The authors declare that the research was conducted in the absence of any commercial or financial relationships that could be construed as a potential conflict of interest.

## Publisher's Note

All claims expressed in this article are solely those of the authors and do not necessarily represent those of their affiliated organizations, or those of the publisher, the editors and the reviewers. Any product that may be evaluated in this article, or claim that may be made by its manufacturer, is not guaranteed or endorsed by the publisher.
