# Supplementary material for: Prevalence and Demographic Risk Factors of Mycobacterium tuberculosis Infections in Captive Asian Elephants (Elephas maximus) Based on Serological Assays
Source: Front Vet Sci. 2021 Nov 2;8:713663. doi: 10.3389/fvets.2021.713663 (PMC8630616; doi:10.3389/fvets.2021.713663)
Supplement: Supplementary file 1 [file Table_1.docx]

Table S1. Pairwise correlations of test results between the four serological tests. Pearson’s correlation between ELISAs (S/P ratios) and Spearman correlation of Stat-Pak assay (pos/neg) with 3 ELISAs.

| Serological Test |  | ESAT6 | CFP10 | MPB83 |
| --- | --- | --- | --- | --- |
| TB Stat-Pak |  | 0.098 | 0.191 | 0.190 |
| ESAT6 |  |  | 0.417 | 0.377 |
| CFP10 |  |  |  | 0.642 |
