# Supplementary material for: Prevalence and Demographic Risk Factors of Mycobacterium tuberculosis Infections in Captive Asian Elephants (Elephas maximus) Based on Serological Assays
Source: Front Vet Sci. 2021 Nov 2;8:713663. doi: 10.3389/fvets.2021.713663 (PMC8630616; doi:10.3389/fvets.2021.713663)
Supplement: Supplementary file 2 [file Table_2.docx]

Table S2. Distribution (frequency and percentages) of dichotomized S/P ratios for each ELISA test per predicted TB serology test outcome using the estimated cut-off value (Table 3) for a positive test for sera of 708 elephants.

* Latent Class Analysis was used to predict TB serology test outcome per individual using four serological tests (ESAT6, CFP10, MPB83 and TB Stat-Pak).

n = number, % = percentage within predicted TB serology test outcome per test

|  |  |  | Predicted serological TB status* | | |
| --- | --- | --- | --- | --- | --- |
|  |  |  | Positive  106 (15.0%) | Inconclusive  357 (50.4%) | Negative  245 (34.6%) |
| Serological test | Single test status | Total n (%) | n (%) | n (%) | n (%) |
| ESAT6 | Negative | 449 (63.4) | 35 (33.0) | 182 (51.0) | 232 (84.7) |
|  | Positive | 259 (36.6) | 71(67.0) | 175 (49.0) | 13 (5.3) |
| CFP10 | Negative | 521 (73.6) | 9 (8.5) | 267 (74.5) | 245 (100) |
|  | Positive | 187 (26.4) | 97 (91.5) | 90 (25.2) | 0 (0) |
| MPB83 | Negative | 520 (73.4) | 13 (12.3) | 262 (73.4) | 245 (100) |
|  | Positive | 188 (26.6) | 93 (87.7) | 95 (26.6) | 0 (0) |
| TB Stat-Pak | Negative | 668 (94.4) | 83 (78.3) | 243 (96.1) | 242 (98.8) |
|  | Positive | 40 (5.6) | 23 (21.7) | 14 (3.9) | 3 (1.2) |
