# Supplementary material for: Prevalence and Demographic Risk Factors of Mycobacterium tuberculosis Infections in Captive Asian Elephants (Elephas maximus) Based on Serological Assays
Source: Front Vet Sci. 2021 Nov 2;8:713663. doi: 10.3389/fvets.2021.713663 (PMC8630616; doi:10.3389/fvets.2021.713663)
Supplement: Supplementary file 3 [file Table_3.docx]

Table S3. Numbers (percentages) of elephants assigned to each of the three TB serology test outcomes predicted by Latent Class Analysis per demographic variable.

n= number; *Estimated individual percentages of TB serology test outcomes by applied LCA model.

|  |  |  | Predicted TB serology test outcomes | | |
| --- | --- | --- | --- | --- | --- |
|  |  |  | Positive | Inconclusive | Negative |
|  | Category | Total (%) | n (%) | n (%) | n (%) |
| Variable | Total | 708 (100) | 106 (15)* | 357 (50)* | 245 (35)* |
| Sex | Female | 535 (76) | 79 (15) | 275 (51) | 181 (34) |
|  | Male | 173 (24) | 27 (16) | 82 (47) | 64 (37) |
| BCS | 1-2 | 11 (2) | 3 (27) | 7 (64) | 1 (9) |
|  | 3-4 | 25 (4) | 3 (12) | 12 (48) | 10 (40) |
|  | 5-6 | 643 (91) | 99 (15) | 325 (51) | 219 (34) |
|  | 7-8 | 27 (4) | 0 (0) | 12 (44) | 15 (56) |
|  | 9-11 | 2 (0) | 1 (50) | 1 (50) | 0 (0) |
| Age (y) | 1-3 | 23 (3) | 3 (13) | 7 (30) | 13 (57) |
|  | 4-10 | 101 (14) | 7 (12) | 31 (53) | 21 (36) |
|  | 11-50 | 587 (83) | 85 (14) | 305 (52) | 197 (34) |
|  | 51-60 | 29 (4) | 8 (28) | 11 (38) | 10 (34) |
|  | > 61 | 100 (14) | 3 (30) | 3 (30) | 4 (40) |
| Working | 0 | 10 (1) | 2 (20) | 4 (40) | 4 (40) |
| Time (hr/d) | 2 | 1 (0) | 0 (0) | 0 (0) | 1 (100) |
|  | 3 | 47 (7) | 14 (30) | 21 (45) | 12 (26) |
|  | 7 | 650 (92) | 90 (14) | 332 (51) | 228 (35) |
| Feed type | Natural - human | 422 (60) | 57 (14) | 226 (54) | 139 (33) |
|  | Natural | 102 (14) | 17 (17) | 44 (43) | 41 (40) |
|  | Human | 184 (26) | 32 (17) | 87 (47) | 65 (35) |
| Management | Extensive | 215 (30) | 29 (13) | 100 (47) | 86 (40) |
| system | Intensive | 493 (70) | 77 (16) | 257 (52) | 159 (32) |
| Camp size (n) | 1-9 | 161 (23) | 29 (18) | 75 (47) | 57 (35) |
|  | 10-30 | 244 (34) | 45 (18) | 129 (53) | 70 (29) |
|  | 31-50 | 120 (17) | 7 (6) | 72 (60) | 41 (34) |
|  | > 51 | 183 (26) | 25 (14) | 81 (40) | 77 (42) |
| Region | Central | 18 (3) | 4 (22) | 12 (67) | 2 (11) |
|  | East | 129 (18) | 11 (9) | 81 (63) | 37 (29) |
|  | North | 269 (38) | 16 (6) | 115 (43) | 138 (51) |
|  | Northeast | 10 (1) | 5 (50) | 3 30) | 2 (20) |
|  | South | 160 (23) | 31 (19) | 83 (52) | 46 (29) |
|  | West | 122 (17) | 39 (32) | 63 (52) | 20 (16) |
