# Supplementary material for: Prevalence and Demographic Risk Factors of Mycobacterium tuberculosis Infections in Captive Asian Elephants (Elephas maximus) Based on Serological Assays
Source: Front Vet Sci. 2021 Nov 2;8:713663. doi: 10.3389/fvets.2021.713663 (PMC8630616; doi:10.3389/fvets.2021.713663)

**SUPPLEMENTARY MATERIAL**

**Supplemental Figure S1.**

Receiver Operation Curves (ROC) to estimate the cut-off value, sensitivity and specificity for each test based on the predicted TB state (“TRUTH”) using the LCA-method (4 tests).

Receiver Operation Curves (ROC) for four serological assays (ESAT6, CFP10 and MPB83 ELISAs and TB Stat-Pak) based on a Latent Class Analysis to predict three TB serology test outcomes. The predicted *positive* outcome was used as the *true* TB serology test outcome for the ROC analysis.


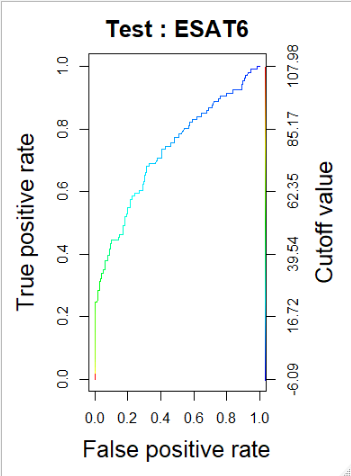

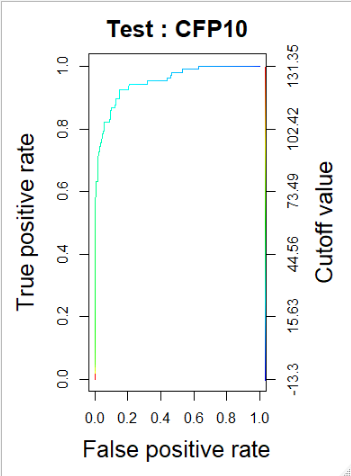

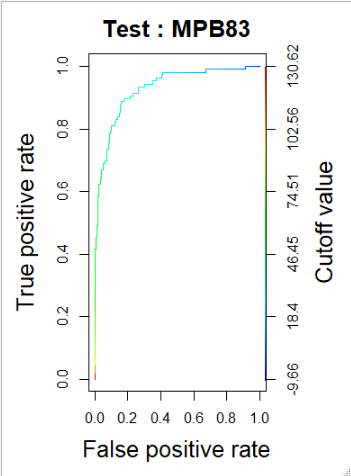

Supplement: Supplementary file 4 [file Data_Sheet_1.docx]
