# Supplementary material for: Prevalence and Demographic Risk Factors of Mycobacterium tuberculosis Infections in Captive Asian Elephants (Elephas maximus) Based on Serological Assays
Source: Front Vet Sci. 2021 Nov 2;8:713663. doi: 10.3389/fvets.2021.713663 (PMC8630616; doi:10.3389/fvets.2021.713663)
Supplement: Supplementary file 5 [file Data_Sheet_2.docx]

**Supplemental Figure S2.** Density plots of S/P ratios of ELISA tests: (A) ESAT6, (B) CFP10 and (C) MPB83 respectively. Two vertical lines indicate estimated cut-off values for separating negative and inconclusive samples (left) and between inconclusive and positive samples (right). n=708.


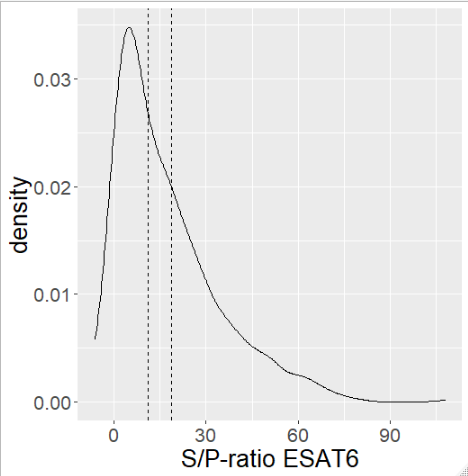

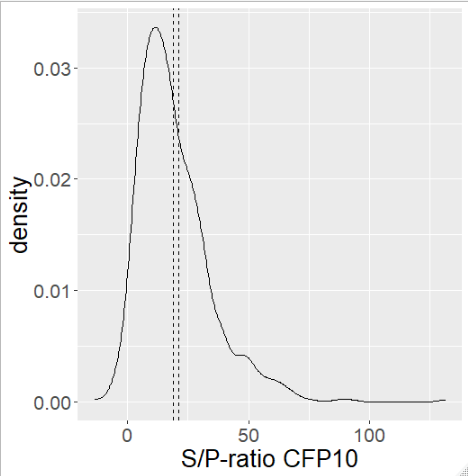

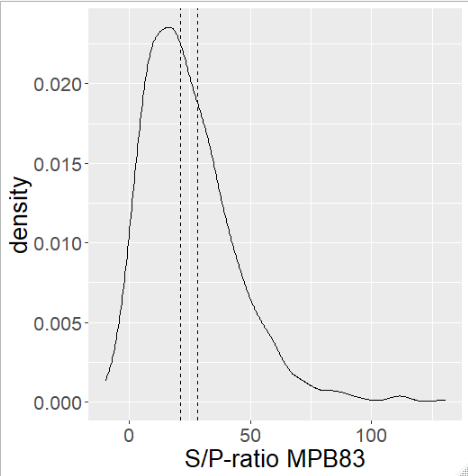


(A) (B) (C)
